# Phase-dependent nutrient requirements of broiler breeder hens for day-old chick production in a field study using interpretable machine learning

**DOI:** 10.1016/j.psj.2026.107028

**Published:** 2026-04-29

**Authors:** Atefeh Jamshasb, Majid Mottaghitalab, Hamed Ahmadi

**Affiliations:** a Department of Animal Science, Faculty of Agricultural Sciences, University of Guilan, Rasht, Iran; b Institute of Animal Science, University of Hohenheim, Stuttgart, Germany

**Keywords:** Broiler breeder, Nutrient requirement, Machine learning, Big data

## Abstract

Day-old chick output from broiler breeder flocks is a key determinant of efficiency in the global poultry industry and is highly sensitive to breeder nutrition. This study aimed to quantify phase-dependent requirements of metabolizable energy and digestible amino acids (lysine, methionine, and threonine) using a large-scale commercial dataset and an interpretable modeling framework. Data from 2,685 flock-week observations (2016–2022) were analyzed across three production phases: early (26–32 weeks), peak (33–50 weeks), and late (51–62 weeks). Nutrient–response relationships were modeled using both classical regression and a single-neuron artificial neural network with a hyperbolic tangent activation, enabling flexible, nonlinear dose–response estimation while preserving biological interpretability. Nutrient requirements were defined as the intake required to achieve 95% of the asymptotic response and were estimated with bootstrap-derived confidence intervals. The ME requirements were highest in early production (453 kcal/d) and declined slightly during peak (449 kcal/d) and late production (448 kcal/d); the narrow numerical range across phases warrants cautious interpretation despite statistical significance. In contrast, amino acid requirements followed reproductive intensity, peaking during mid-production (digestible lysine 1.03 g/d, methionine 0.68 g/d, threonine 0.80 g/d) and decreasing thereafter. The nonlinear modeling approach consistently captured saturating responses that were not adequately described by linear models. These results demonstrate that nutrient requirements in broiler breeders are phase-dependent and can be effectively quantified using interpretable machine learning applied to field data. The proposed framework provides a practical tool for deriving biologically meaningful requirement estimates and supports more precise, phase-specific feeding strategies in commercial production systems.

## Introduction

Broiler breeder hen requirements for metabolizable energy (ME) and digestible amino acids—particularly lysine (dLys), methionine (dMet), and threonine (dThr)—vary across the laying cycle (Ekmay et al., 2013). Accurate estimation of these requirements is essential to maximize the number of marketable day-old chicks, a critical economic and welfare outcome in commercial production (Chang et al., 2016; Cerrate et al., 2019).

Nutrient-response approaches consider one nutrient at a time, typically rely on nonlinear regression models such as linear, quadratic, or logistic functions (Rodehutscord and Pack, 1999). While interpretable, these models impose rigid functional forms and often fail to capture complex response patterns, especially when datasets are sparse or noisy, as is common in on-farm data. Machine learning (ML) offers greater flexibility for modeling nonlinear biological responses, but its adoption in nutrition has been limited by concerns over interpretability and “black-box” predictions (Ching et al., 2018).

Recent studies have demonstrated that minimalist, interpretable ML architecture (Rudin, 2019; Mignan and Broccardo, 2019; Murdoch et al., 2019) such as a single artificial neuron with a hyperbolic tangent activation can reconcile flexibility with nutritional insight (Ahmadi and Rodehutscord, 2025b). This approach produces smooth, saturating response curves and enables analytically derived metrics, such as nutrient requirements at 95% of maximum performance, with robust uncertainty estimates obtained via bootstrap resampling (Carpenter and Bithell, 2000).

In this study, we applied an interpretable ML framework to a large, real-world dataset from commercial broiler breeder flocks in northern Iran. We modeled the relationship between daily nutrient intakes (ME, dLys, dMet, and dThr) and a reproductive response (number of chicks per hen per week) across three production phases: early, mid, and late. Our objectives were to quantify how nutrient requirements shift with age and to provide stage-specific feeding recommendations that reflect practical field conditions.

## Materials and methods

All experimental procedures were approved by the Research Ethics Committee on Animal Use at the University of Guilan (ID: IR. GUILAN.REC. ETHICS-2402-1076).

### Data collection and preprocessing

Data were collected from broiler breeder farms and hatcheries of poultry production companies in Guilan Province, northern Iran (37°16′28″N, 49°35′20″E) over the period 2016–2022. All birds in the study were Ross 308 parent stock (Aviagen, 2021). The dataset includes bird age (weeks), daily feed intake, dietary composition (ME, dLys, dMet, and dThr), and the outcome variable: the number of chicks produced per hen per week.

The raw dataset initially comprised 3,091 records. Dietary intakes of digestible amino acids were recalculated using daily feed intake and their respective digestibility coefficients, while ME intake was calculated directly from feed intake. For consistency, all units were standardized across the database (e.g., energy expressed in kcal/kg, amino acids as g/day/bird). Missing values were identified and flagged. Outliers were identified and controlled through graphical inspection (Aguinis et al., 2013) and comparison with physiologically reasonable ranges. After preprocessing, 2,685 records remained and were categorized into three production phases—early (26–32 weeks, n = 515), mid (33–50 weeks, n = 1,471), and late (51–62 weeks, n = 699) egg production. This cleaned dataset was used as the working database for subsequent analyses.

### Model fitting

Nutrient–response relationships in broiler breeders were modeled using a single artificial neuron with a hyperbolic tangent (tanh) activation function, following the approach developed by (Ahmadi and Rodehutscord, 2025b). This framework captures monotonic, saturating responses typical of essential nutrients (Morgan et al., 1975) and allows biologically interpretable parameter estimation. The model is expressed as:

Response=Atanh(cNutrient+b)+B

where Nutrient and Response are the input and output of the neuron, respectively; A, c, b, and B are trainable parameters estimated from the data. The asymptotic response is given by $\text{Respons}e_{\infty }=A+B$, which represents the physiological ceiling under unlimited (infinity) nutrient supply. Nutrient requirements to achieve 95% of the asymptotic response ($\text{Re}q_{95\%}$) were calculated using:

$$\text{Re}q_{95\%}=\frac{arctanh\left(0.95-0.05\frac{B}{A}\;\right)-\;b}{c}$$

The methodology incorporated Bayesian regularization (Dan Foresee and Hagan, 1997) to prevent overfitting, and non-parametric bootstrap resampling (100 iterations) to estimate uncertainty (Carpenter and Bithell, 2000) and 95% confidence interval (CI) for all parameters and nutrient requirements. All analyses were performed using the MATLAB® (MATLAB R2024b, 2024) based *NutriCurvist* software, which automates model fitting, parameter estimation, and the calculation of nutritional requirements. The software is openly available at (Ahmadi and Rodehutscord, 2025a) and GitHub https://github.com/animalgorithm/NutriCurvist_ver_01.

## Results

**Fig. 1:**
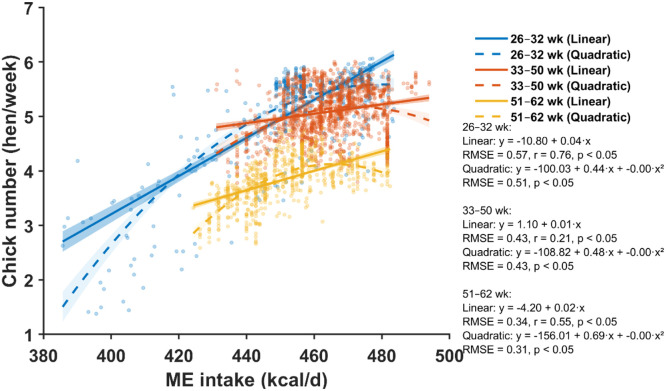
Relationship between daily metabolizable energy (ME) intake and chick number across three production periods (26–32, 33–50, and 51–62 weeks of age). Scatter points represent individual hen-week observations. Solid and dashed lines show linear and quadratic regressions for each age period, respectively, with shaded areas indicating 95% confidence bands. Regression equations, root mean square error (RMSE), *p*-values for model terms, and the linear correlation coefficient (*r*) are provided for each age period.

**Fig. 2:**
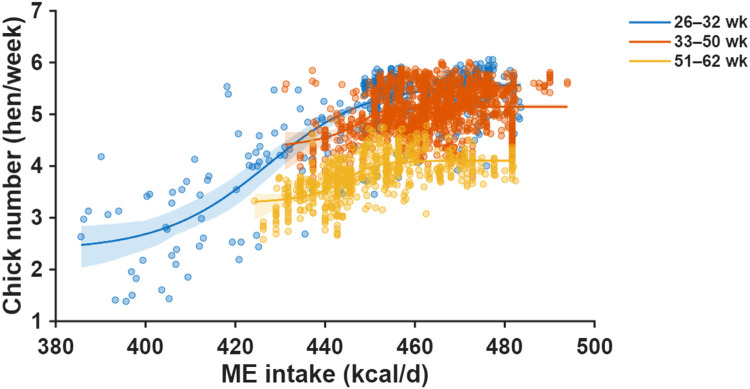
Nonlinear dose–response relationships between daily metabolizable energy (ME) intake and chick number fitted using a single-neuron artificial neural network (ANN) for three production periods (26–32, 33–50, and 51–62 weeks of age). Scatter points show individual observations, and the ANN-predicted curves illustrate the age-specific nonlinear response patterns. Shaded areas represent 95% confidence intervals of the ANN-predicted mean response.

**Fig. 3:**
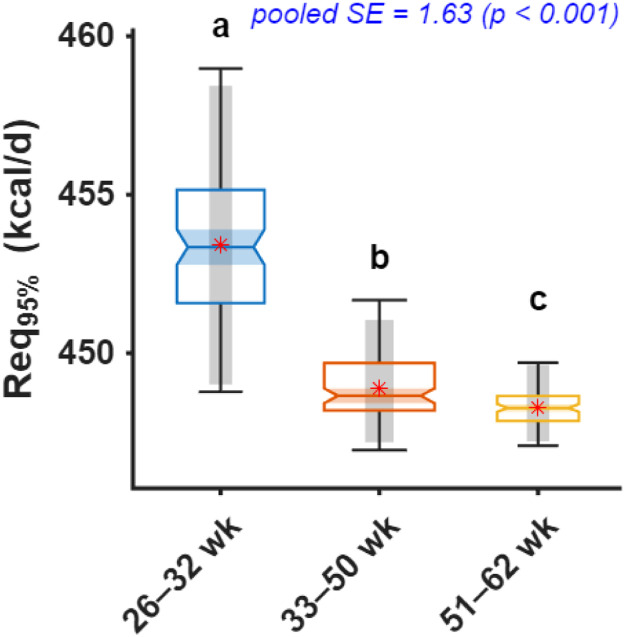
Comparison of daily metabolizable energy intake required to achieve 95% of maximum chick number output (Req_95%_) across production periods. Notched boxplots display the median, interquartile range, and variability of bootstrap estimates, with means shown as red stars. Different letters above boxes indicate significant differences among age periods based on bootstrap-derived post hoc comparisons (p < 0.05). For methodological details, see Ahmadi and Rodehutscord (2025b).

**Fig. 4:**
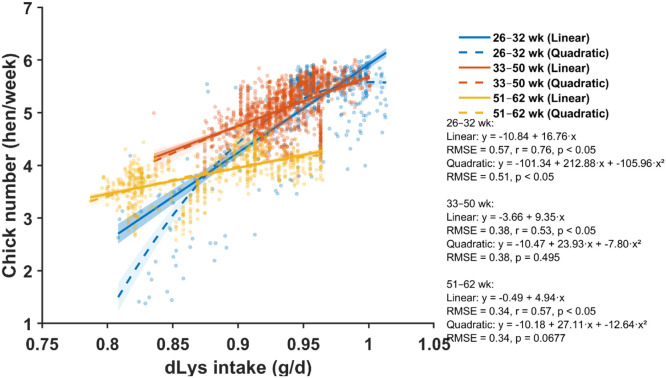
Relationship between daily digestible lysine (dLys) intake and chick number across three production periods (26–32, 33–50, and 51–62 weeks of age). Scatter points represent individual hen-week observations. Solid and dashed lines show linear and quadratic regressions for each age period, respectively, with shaded areas indicating 95% confidence bands. Regression equations, root mean square error (RMSE), *p*-values for model terms, and the linear correlation coefficient (*r*) are provided for each age period.

**Fig. 5:**
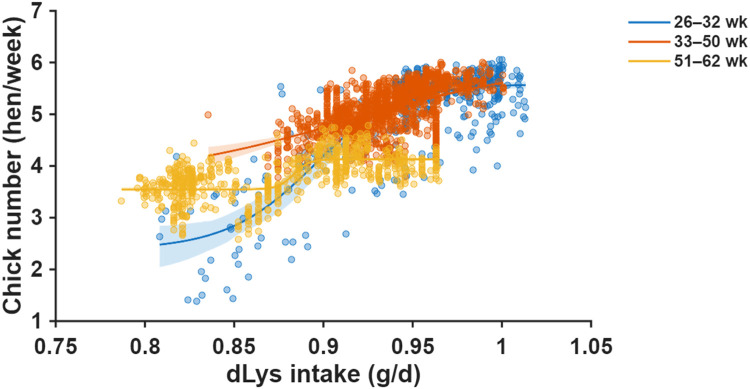
Nonlinear dose–response relationships between daily digestible lysine (dLys) intake and chick number fitted using a single-neuron artificial neural network (ANN) for three production periods (26–32, 33–50, and 51–62 weeks of age). Scatter points show individual observations, and the ANN-predicted curves illustrate the age-specific nonlinear response patterns. Shaded areas represent 95% confidence intervals of the ANN-predicted mean response.

**Fig. 6:**
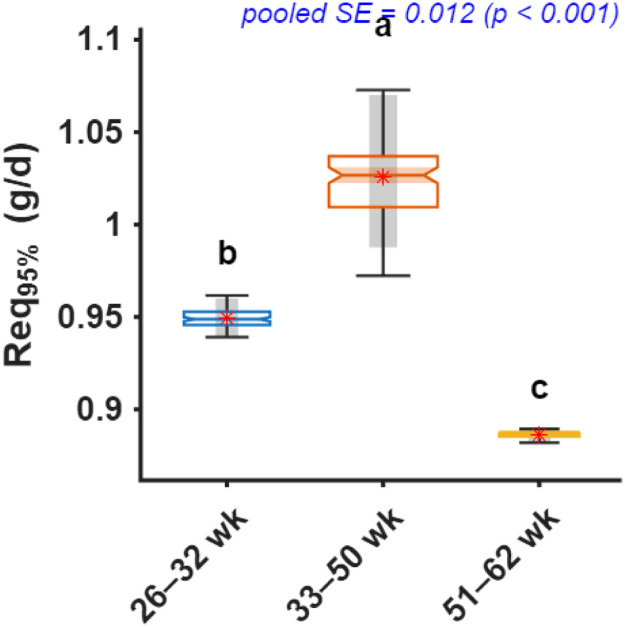
Comparison of daily digestible lysine intake required to achieve 95% of maximum chick number output (Req_95%_) across production periods. Notched boxplots display the median, interquartile range, and variability of bootstrap estimates, with means shown as red stars. Different letters above boxes indicate significant differences among age periods based on bootstrap-derived post hoc comparisons (p < 0.05).

**Fig. 7:**
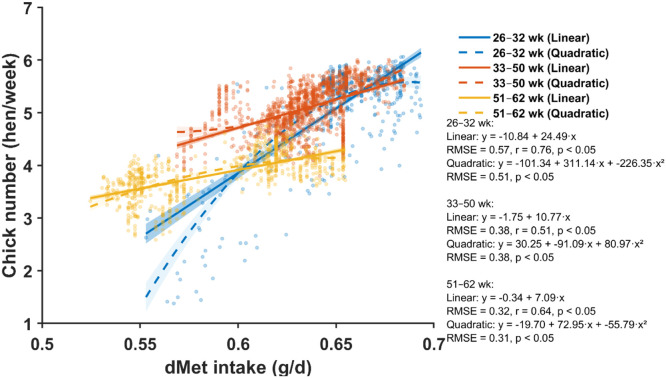
Relationship between daily digestible methionine (dMet) intake and chick number across three production periods (26–32, 33–50, and 51–62 weeks of age). Scatter points represent individual hen-week observations. Solid and dashed lines show linear and quadratic regressions for each age period, respectively, with shaded areas indicating 95% confidence bands. Regression equations, root mean square error (RMSE), *p*-values for model terms, and the linear correlation coefficient (*r*) are provided for each age period.

**Fig. 8:**
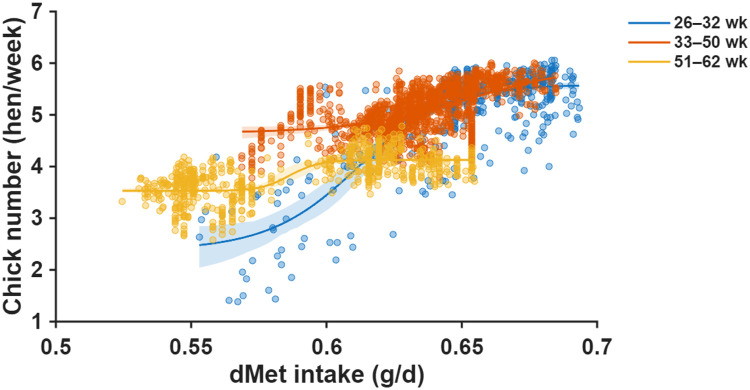
Nonlinear dose–response relationships between daily digestible methionine (dMet) intake and chick number fitted using a single-neuron artificial neural network (ANN) for three production periods (26–32, 33–50, and 51–62 weeks of age). Scatter points show individual observations, and the ANN-predicted curves illustrate the age-specific nonlinear response patterns. Shaded areas represent 95% confidence intervals of the ANN-predicted mean response.

**Fig. 9:**
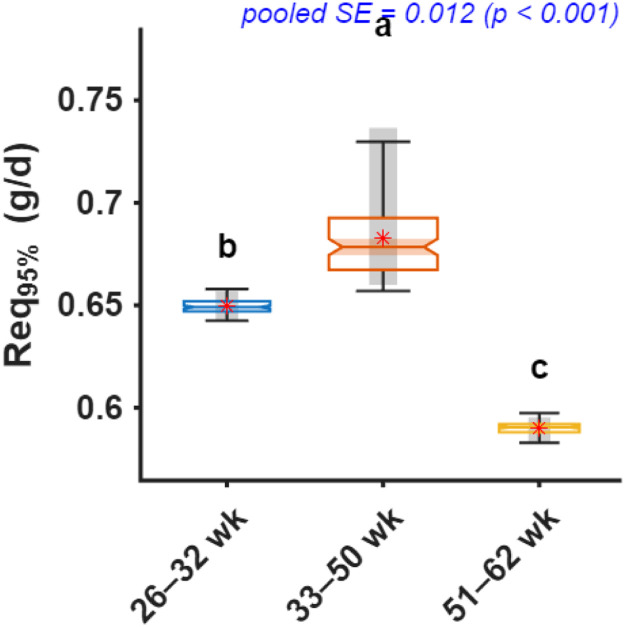
Comparison of daily digestible methionine intake required to achieve 95% of maximum chick number output (Req_95%_) across production periods. Notched boxplots display the median, interquartile range, and variability of bootstrap estimates, with means shown as red stars. Different letters above boxes indicate significant differences among age periods based on bootstrap-derived post hoc comparisons (p < 0.05).

**Fig. 10:**
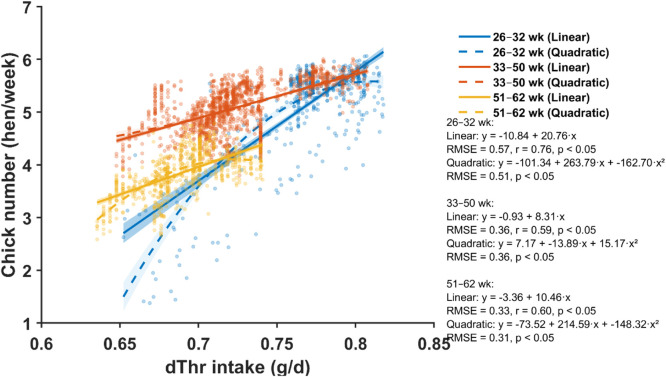
Relationship between daily digestible threonine (dThr) intake and chick number across three production periods (26–32, 33–50, and 51–62 weeks of age). Scatter points represent individual hen-week observations. Solid and dashed lines show linear and quadratic regressions for each age period, respectively, with shaded areas indicating 95% confidence bands. Regression equations, root mean square error (RMSE), *p*-values for model terms, and the linear correlation coefficient (*r*) are provided for each age period.

**Fig. 11:**
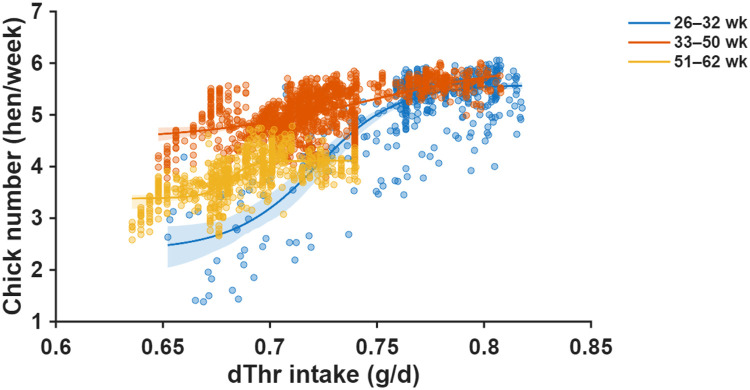
Nonlinear dose–response relationships between daily digestible threonine (dThr) intake and chick number fitted using a single-neuron artificial neural network (ANN) for three production periods (26–32, 33–50, and 51–62 weeks of age). Scatter points show individual observations, and the ANN-predicted curves illustrate the age-specific nonlinear response patterns. Shaded areas represent 95% confidence intervals of the ANN-predicted mean response.

**Fig. 12:**
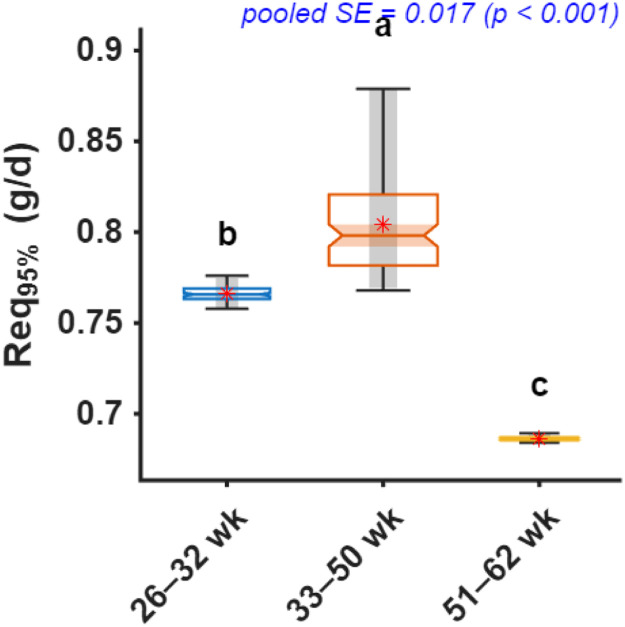
Comparison of daily digestible threonine intake required to achieve 95% of maximum chick number output (Req_95%_) across production periods. Notched boxplots display the median, interquartile range, and variability of bootstrap estimates, with means shown as red stars. Different letters above boxes indicate significant differences among age periods based on bootstrap-derived post hoc comparisons (p < 0.05).

A total of 12 figures (Fig. 1, Fig. 2, Fig. 3, Fig. 4, Fig. 5, Fig. 6, Fig. 7, Fig. 8, Fig. 9, Fig. 10, Fig. 11, Fig. 12) summarize the key findings of this study, depicting the main patterns in nutrient intake, chick output, and the resulting requirement estimates. To improve the clarity of the requirement comparisons, we used modified box plots (McGill et al., 1978) in Figs. 3, 6, 9, and 12. These plots display the median, interquartile range, and whiskers, with notches indicating an approximate 95% CI around the median. We also added the mean and its bootstrap-based 95% CI, which serve as the primary indicators of central tendency and uncertainty. Additional details on the construction and interpretation of these enhanced box plots are provided in Fig. 2 of Ahmadi and Rodehutscord (2025b).

### Metabolizable energy

The relationship between daily ME intake and chick production exhibited distinct age-dependent patterns across the three production phases (Fig. 1, Fig. 2). Linear regression analysis revealed relatively strong positive correlations in the early phase (26–32 weeks; r = 0.76, p < 0.05), which weakened in the mid-phase (33–50 weeks; r = 0.21, p < 0.05) and improved again in the late phase (51–62 weeks; r = 0.55, p < 0.05). Quadratic models improved fit over linear models, particularly in the early phase [root mean square error (RMSE) reduced from 0.57 to 0.51.

Single-neuron ANN modeling revealed nonlinear, saturating response curves across all production periods (Fig. 2). Model performance, evaluated using RMSE, varied across production phases, with values of 0.41, 0.12, and 0.20 hen/week in the early, mid, and late phases, respectively.

Bootstrap-derived estimates indicated that ME intake required to achieve 95% of maximum chick output was significantly higher (p < 0.001) in the early phase compared with the mid and late phases (Fig. 3). Hens aged 26–32 weeks required 453.4 kcal/d ME (95% CI: 449.0–458.4), whereas requirements declined to 448.9 kcal/d (447.2–451.1) and 448.3 kcal/d (447.2–449.6) during the peak (33–50 weeks) and late (51–62 weeks) phases, respectively.

### Lysine

Daily dLys intake showed positive associations with chick production across all phases, although correlation patterns varied (Fig. 4). Linear correlations were strong and consistent in the early (r = 0.76, p < 0.05), mid (r = 0.53, p < 0.05), and late phases (r = 0.57, p < 0.05). Quadratic models improved fit over linear models only in the early phase (RMSE reduced from 0.57 to 0.51) but provided no improvement during mid-production (quadratic p = 0.495). Single-neuron ANN modeling captured nonlinear, saturating response curves across all production phases (Fig. 5).

Bootstrap-derived estimates indicated that early-phase hens required 0.95 g/d dLys (95% CI: 0.94–0.96), which increased significantly to 1.03 g/d (0.99–1.07) during peak production (33–50 weeks) and declined to 0.89 g/d (0.88–0.89) in the late phase (Fig. 6).

### Methionine

The relationship between dMet intake and chick production showed positive correlations across production phases (Fig. 7). Linear correlations were consistently high: r = 0.76 (p < 0.05) in early production, r = 0.51 (p < 0.05) in mid-phase, and r = 0.64 (p < 0.05) in late phase. Quadratic models improved fit over linear models only in the early phase (RMSE reduced from 0.57 to 0.51) and provided no improvement during the mid and late phases.

Single-neuron ANN modeling captured smooth, nonlinear response curves for dMet across all production periods (Fig. 8), achieving RMSE values of 0.41, 0.20, and 0.21 hen/week in the early, mid, and late phases, respectively—performance nearly identical to that observed for dLys.

Bootstrap-derived estimates indicated that hens aged 26–32 weeks required 0.65 g/d dMet (95% CI: 0.64–0.66), which increased to 0.68 g/d (0.66–0.74) during peak production and declined to 0.59 g/d (0.58–0.60) in late production (Fig. 9).

### Threonine

Daily dThr intake demonstrated positive linear relationships with chick production across all phases (Fig. 10), with correlation coefficients of r = 0.76 (p < 0.05), r = 0.59 (p < 0.05), and r = 0.60 (p < 0.05) for early, mid, and late phases, respectively. Quadratic models improved fit over linear models only in the early phase (RMSE reduced from 0.57 to 0.51) and provided no improvement during the mid and late phases.

ANN-derived dose-response curves for threonine exhibited similar nonlinear saturation patterns as the other amino acids (Fig. 11), with RMSE values of 0.408, 0.212, and 0.206 hen/week for early, mid, and late phases, respectively. Threonine requirements mirrored the age-dependent trajectory observed for lysine and methionine (Fig. 12), with early-phase requirements estimated at 0.77 g/d dThr (0.76–0.77), rising to 0.80 g/d (0.77–0.88) during peak production, and declining to 0.69 g/d (0.68–0.69) in the late phase.

## Discussion

This study is based on observational field data collected under commercial production conditions and therefore reflects the inherent variability of real-world systems. Factors such as farm management practices, environmental conditions (e.g., temperature and housing), health status, and genetic heterogeneity among flocks were not explicitly controlled or modeled. Consequently, the identified nutrient–response relationships should be interpreted as population-level associations rather than strictly causal effects. At the same time, this variability represents a key strength of the study, as it enhances the practical relevance and generalizability of the findings to commercial settings. The ability to extract consistent and biologically meaningful patterns from heterogeneous field data is a central challenge in applied nutritional research. Recent work has emphasized the importance of robust modeling frameworks for decomposing complex, real-world systems and deriving reliable insights under operational conditions (Bakirci, 2024). In this context, the combination of large-scale commercial data with interpretable modeling provides a promising approach to bridge the gap between controlled experimental findings and practical feeding strategies.

The definition of production phases (early, peak, and late) follows common industry practice and reflects the biological progression of broiler breeder hens. However, these phase boundaries represent simplified categorizations of a continuous process. Small shifts in phase cutoffs would not be expected to materially alter the overall trends observed, as nutrient–response relationships were modeled continuously within each phase. The phase-based stratification was primarily adopted to facilitate biological interpretation and alignment with practical feeding strategies.

Nutrient requirements in this study were defined as the intake required to achieve 95% of the asymptotic response (Req95%), representing a pragmatic balance between maximizing biological performance and avoiding excessive nutrient supply. While this threshold is widely used in nutritional modeling, it is inherently operational rather than absolute. Alternative thresholds (e.g., 90% or 97%) would result in different absolute requirement values; however, the relative differences among production phases and the overall response patterns would remain largely consistent. Thus, the choice of threshold primarily influences the degree of conservatism in feeding recommendations rather than the underlying biological interpretation.

The pattern of ME requirements revealed a slight inverse relationship with age and production intensity. Early-phase hens (26–32 weeks) required the highest ME intake (453.4 kcal/d) to achieve 95% of maximum chick output, despite lower absolute production levels. Requirements then declined modestly to 448.9 kcal/d during peak production (33–50 weeks) and remained stable at 448.3 kcal/d in late production (51–62 weeks). Although these differences were statistically significant, their magnitude was small (<1.2%) and should be interpreted with caution in practical feeding applications. Although statistically significant, the differences in ME requirements across production phases were relatively small in absolute terms. Therefore, these differences should be interpreted with caution in practical applications. This seemingly counterintuitive pattern can be explained by age-related shifts in energy partitioning. During early production, hens are still undergoing skeletal development, body protein deposition, and reproductive system maturation. These concurrent processes increase maintenance, and growth-related energy demands relative to reproductive output, resulting in a higher energy requirement per unit of chick production (Sakomura, 2004). As hens progress into peak production, somatic growth declines and a greater proportion of dietary energy is directed toward egg formation and reproductive processes, thereby improving energetic efficiency despite higher absolute output. Our estimated peak-phase ME intake requirement of 448.9 kcal/d was relatively lower than the Ross 308 parent stock nutrition recommendation of 468 kcal/d at peak production (Aviagen, 2021). This 4.1% difference may reflect several factors inherent to field conditions versus breeder company performance objectives. From a practical perspective, the small numerical differences in ME requirements across phases suggest that energy supply in commercial systems is relatively stable, with only fine adjustments required.

In contrast to energy, digestible amino acid requirements closely mirrored production intensity across the laying cycle. Lysine, methionine, and threonine all exhibited peak requirements during mid-production (33–50 weeks)—when egg mass and chick numbers are highest—followed by significant declines in late lay. Peak-phase requirements were estimated at 1.03 g/d dLys, 0.68 g/d dMet, and 0.80 g/d dThr, representing increases of 8.4%, 4.6%, and 3.9%, respectively, relative to early production. Our peak-phase lysine requirement of 1.03 g/d aligned very closely with the Ross 308 Parent Stock nutrition guideline, which recommends 1.036 g/d digestible lysine at peak production (Aviagen, 2021). The estimated methionine requirement of 0.68 g/d during peak production was approximately 7% higher than the Ross 308 specification of 0.635 g/d. In contrast, our threonine requirement of 0.80 g/d was about 14% lower than the Ross 308 recommendation of 0.919 g/d (Aviagen, 2021). Our peak-phase estimates for dLys, dMet, and dThr were also consistently higher than those reported by (Ekmay et al., 2013), who identified optimal intakes of 0.916 g/d dLys, 0.424 g/d dMet, and 0.613 g/d dThr for maximizing egg mass and fertility at peak lay. In comparison, our estimates of 1.03 g/d Lys (+12%), 0.68 g/d Met (+60%), and 0.80 g/d Thr (+31%) indicate greater amino acid demands in the current dataset. These discrepancies may reflect differences in genetic strain productivity and target performance traits, as well as differences in the modeling strategy and statistical criteria used to define optimal intake. Notably, our estimates align more closely with contemporary breeder recommendations (Aviagen, 2021), suggesting that amino acid requirements at peak production may have changed over decade due to continued development.

The uniform decline in amino acid requirements during late production (51–62 weeks)—when dLys, dMet, and dThr requirements fell to 0.89, 0.59, and 0.69 g/d, respectively—reflects reduced egg output and potentially altered body protein turnover in aging hens. Maintaining peak-phase amino acid levels during late production would represent biological oversupply, increasing nitrogen excretion and feed costs without corresponding improvements in chick yield. These findings support phase-specific amino acid programming rather than uniform feeding throughout lay.

The single-neuron ANN framework demonstrated superior fit compared to traditional linear and quadratic regression models, particularly for capturing saturating nutrient responses typical of essential nutrients. RMSE values for the ANN models consistently outperformed linear regression across all nutrients and phases, with the greatest advantage observed during early production (e.g., RMSE 0.41 vs. 0.57 for ME in weeks 26–32). Notably, quadratic models improved fit only in the early phase and offered no advantage during peak or late production, where nonlinear ML approaches were necessary to characterize diminishing marginal returns at high nutrient intakes.

The interpretability of the tanh-based neuron—achieved through analytically derived requirement estimates and bootstrap-based confidence intervals—addresses the primary criticism of ML in nutritional science: the "black box" problem (Ching et al., 2018). Unlike deep learning architectures that sacrifice transparency for flexibility, this minimalist approach preserves biological interpretability (Murdoch et al., 2019) while accommodating complex dose-response relationships. The bootstrap-derived uncertainty estimates (95% CI) provide statistically rigorous bounds for on-farm decision-making, a critical advantage over point estimates from conventional models. These CI intervals reflect the variability inherent in the dataset and the stability of the model-derived parameters. From a practical standpoint, narrower confidence intervals indicate greater certainty in requirement estimates, whereas wider intervals suggest increased variability and the need for more cautious application. In commercial feeding decisions, such uncertainty ranges can be used to guide risk management strategies, for example by selecting target nutrient levels that account for both expected performance and acceptable variability.

This study demonstrates the feasibility of applying advanced analytical methods to real-world farm data—a critical step toward precision livestock farming. The dataset, spanning six years and multiple commercial flocks with inherent management variability, represents the heterogeneity typical of production systems. Despite this "noisy" data environment, the ML framework extracted robust nutrient-response relationships and biologically coherent requirement estimates. This suggests that large-scale farm databases, when properly curated and analyzed, can refine nutritional recommendations in ways that controlled experimental trials—limited by sample size, genetic uniformity, and environmental standardization—cannot achieve.

The response variable used in this study—chicks produced per hen per week—represents a composite outcome integrating egg production, fertility, and hatchability. While this metric is highly relevant from a production perspective, it may mask differential effects of individual nutrients on its underlying components. For example, certain nutrients may primarily influence egg formation, whereas others may affect embryonic development or hatchability. Therefore, the observed nutrient–response relationships should be interpreted as integrated effects across multiple biological processes.

### Future directions

First, our outcome variable—chicks per hen per week—integrates multiple biological processes (egg production, fertility, hatchability) that may respond differently to individual nutrients. Future work disaggregating these components and optimizing across multiple reproductive objectives simultaneously may benefit from multi-objective ML frameworks (Ahmadi et al., 2023) that explicitly quantify trade-offs between competing response traits (e.g., distinguishing amino acid effects on egg mass vs. embryonic viability). Second, nutrient interactions (e.g., amino acid ratios, energy-to-protein balance) were not explicitly modeled; multivariate ML approaches could capture synergistic or antagonistic effects among dietary components. Future iterations of this approach could incorporate additional covariates (e.g., ambient temperature, body weight trajectories, disease challenges) to further personalize nutrient recommendations at the flock level. Integration with automated feeding systems and real-time production monitoring would enable dynamic adjustment of nutrient supply based on observed performance, moving from static phase-feeding toward truly responsive nutrition.

## Conclusion

Our results show that ME requirements decline modestly with age — varying by less than 6 kcal/d across phases— while amino acid requirements follow reproductive intensity more distinctly, peaking during mid-lay and decreasing thereafter. Based on over 2,600 flock-week observations, tentative phase-specific targets to achieve 95% of maximum chick production are: early production (26–32 weeks) ∼453 kcal/d ME, 0.95 g/d dLys, 0.65 g/d dMet, 0.77 g/d dThr; peak production (33–50 weeks) ∼449 kcal/d ME, 1.03 g/d dLys, 0.68 g/d dMet, 0.80 g/d dThr; and late production (51–62 weeks) ∼448 kcal/d ME, 0.89 g/d dLys, 0.59 g/d dMet, 0.69 g/d dThr. These findings suggest that stepwise adjustment of ME and amino acids according to production phase can improve flock performance, and that interpretable machine learning offers a practical, scalable approach to define nutritional guidelines as production systems evolve.

## CRediT authorship contribution statement

**Atefeh Jamshasb:** Writing – original draft, Resources, Investigation, Formal analysis, Data curation. **Majid Mottaghitalab:** Writing – review & editing, Supervision, Resources, Project administration, Methodology, Investigation, Data curation, Conceptualization. **Hamed Ahmadi:** Writing – review & editing, Supervision, Software, Methodology, Formal analysis.

## Disclosures

The authors declare that the research was conducted in the absence of any commercial or financial relationships that could be construed as a potential conflict of interest. This research did not receive any specific grant from funding agencies in the public, commercial, or not-for-profit sectors
